# Medication preparation program in Liencres Hospital

**DOI:** 10.1192/j.eurpsy.2021.2088

**Published:** 2021-08-13

**Authors:** G. Isidro, A. Bezanilla, M. Galván, Y. Saiz, B. Voces, M. Guitián

**Affiliations:** Ume (hospital De Liencres), Servicio de Psiquiatría, Hospital Universitario Marqués de Valdecilla, Santander, Spain

**Keywords:** Rehabilitation, psychosis, Psychoeducation, medication

## Abstract

**Introduction:**

The need to implement a program of autonomy in the handling of oral medication has been observed at the time of discharge from the hospital.

**Objectives:**

- That the patient is able to know his medication, differentiating between active ingredient and commercial brand. - That the patient is able to interpret the guideline in the electronic prescription. - That the patient is able to prepare his weekly medication autonomously.

**Methods:**

- The doctor in charge indicates the Program in those patients susceptible to benefit of the same and after consensus with the multidisciplinary team. He validates and prints the electronic prescription well in advance. Preferably the patient himself (alone or accompanied by family members or Educators) get their medication and a weekly “polydosis” at a pharmacy office bringing him with him to the Unit. - Occupational Therapy helps the patient interpret the electronic prescription guideline and place the weekly medication in the “polydosis” and works with the patient in forecasting execution tasks of medication needs for outpatient follow-up.

**Results:**

The program is well accepted by patients. None of the patients included so far have had an early relapse.

**Conclusions:**

The program has helped patients interpret the medical indications given mnesical and executive difficulties of patients with severe mental disorder.

**Disclosure:**

No significant relationships.

